# The prevalence and factors associated with sarcopenia among community living elderly with type 2 diabetes mellitus in primary care clinics in Malaysia

**DOI:** 10.1371/journal.pone.0233299

**Published:** 2020-05-20

**Authors:** Shariff-Ghazali Sazlina, Ping Yein Lee, Yoke Mun Chan, Mohamad Shariff A. Hamid, Ngiap Chuan Tan

**Affiliations:** 1 Department of Family Medicine, Faculty of Medicine and Health Sciences, Universiti Putra Malaysia, Serdang, Malaysia; 2 Malaysian Research Institute on Ageing, Universiti Putra Malaysia, Serdang, Malaysia; 3 Department of Nutrition and Dietetics, Faculty of Medicine and Health Sciences, Universiti Putra Malaysia, Serdang, Malaysia; 4 Research Centre of Excellence, Nutrition and Non-communicable Diseases, Faculty of Medicine and Health Sciences, Universiti Putra Malaysia, Serdang, Malaysia; 5 Unit of Sports Medicine, Faculty of Medicine, University of Malaya, Kuala Lumpur, Malaysia; 6 DUKE-NUS Medical School, Singapore, Singapore; Suez Canal University Faculty of Medicine, EGYPT

## Abstract

Sarcopenia is a recognised geriatric syndrome but few studies address its associated factors among elderly with type 2 diabetes mellitus (T2DM) in South East Asia. This study aimed to determine the prevalence of sarcopenia and its associated factors among the elderly with T2DM in public primary care clinics in Malaysia. This study utilised data from a longitudinal study of 506 adults with T2DM aged ≥60 years. Data on socio-demography, clinical and functional status, diet and levels of physical activity (PA) were collected. Sarcopenia was defined using Asian Working Group for Sarcopenia criteria and its associated factors were analysed using multiple logistic regression. The proportion of elderly with T2DM with sarcopenia was 28.5%. Those aged ≥70 years (β = 0.73;OR = 2.07; 95%CI = 1.24, 3.48; p = 0.006), men (β = 0.61; OR = 1.84; 95%CI = 1.12, 3.02; p = 0.017), with ≥10 years duration of diabetes (β = 0.62; OR = 1.85; 95%CI = 1.11, 3.09; p = 0.018), not using insulin sensitizers (β = -1.44; OR = 0.24; 95%CI = 0.08, 0.71; p = 0.010), using less than 5 medications (β = 0.68; OR = 1.98; 95%CI = 1.17, 3.36; p = 0.011), low body mass index (BMI) (β = -2.43; OR = 0.09; 95%CI = 0.05, 0.17; p<0.001), and engaging in low (β = 0.77; OR = 2.15; 95%CI = 1.07, 4.35; p = 0.032) and moderate physical activities (β = 0.80; OR = 2.23; 95%CI = 1.07, 4.66; p = 0.033) were associated with sarcopenia. Factors that predicts sarcopenia such as level of physical activity and body mass index were among the modifiable factors that could be used in developing future strategies to prevent or delay the progression of sarcopenia among elderly with T2DM to improve their health status.

## Introduction

Age-related loss of muscle mass and function is referred to as sarcopenia [1]. Its development has been shown to begin in younger age [2], but often underdetected and undertreated in the clinical practice [3]. Sarcopenia is an important threat to the independence of the elderly and is a recognized geriatric syndrome. Sarcopenia is related to other geriatric syndromes such as the risk of falling, functional impairment, mobility limitations and unfavorable metabolic effects [4–6]. It leads to significant disabilities, poorer quality of life and high healthcare expenditure [7,8].

In 2010, the European Working Group on Sarcopenia in Older People (EWGSOP) proposed an operational definition and diagnostic strategy based on measurements of muscle mass, muscle strength and physical performance [1]. In 2014, the Asian Working Group for Sarcopenia (AWGS) recommended measuring both the handgrip strength and the gait speed as screening tests instead of the gait speed alone. In addition, the cut-off values used by the AWGS for the measurements of muscle mass and strength were lower compared to those recommended by EWGSOP. These modifications were considered necessary as it was proposed that the Asian populations may differ from Caucasians in ethnicities, body size, lifestyles, and cultural backgrounds [4].

The etiologies of sarcopenia is multifactorial and includes inflammation, altered endocrine function, nutritional deficits, physical inactivity and insulin resistance [1]. Patients with type 2 diabetes mellitus (T2DM) experience accelerated muscle loss leading to lower muscle mass [9], and higher rate of sarcopenia than healthy people [10], even after adjusting for age, body mass index, current smoking and other risk factors [9]. T2DM has been shown to be associated with multiple neuromuscular dysfunctions including reduced muscle mass, strength and functional capacity in terms of muscle performance and quality [11,12]. The lean mass and appendicular skeletal muscle mass in elderly with T2DM were lower compared to age-matched normoglycaemic elderly [12]. Also, these elderly also had reduced muscle strength and functional capacity, which were associated with type II muscle fibre atrophy (12) and motor nerve impairment [11]. These deficits in the muscular system among elderly with T2DM may predispose to sarcopenia.

The prevalence of sarcopenia among the elderly with T2DM ranged from 15.7% to 29.3% [13–15]. The differences in the prevalence are due to the different operational definition used for sarcopenia in these studies. The factors related to sarcopenia among elderly with T2DM were increasing of age, men, presence of multimorbidity, diabetic nephropathy, diabetic retinopathy and reduced hip circumference [13–16]. However, the muscle mass loss in elderly men with T2DM was reduced in those who were treated with insulin sensitizers such as metformin [17]. Other factors such as reduced body mass index (BMI)[18,19], physical inactivity [19], poor nutrition [19] and functional impairments[20] have not been reported in elderly with T2DM.

Currently, there are limited studies on factors associated with sarcopenia among elderly with T2DM in South East Asia and none in Malaysia. Identifying the associated factors is imperative as it could lead to a change in policy to improve the function and independence of elderly with T2DM. Moreover, evidence has demonstrated the benefits of exercise and nutrition in improving sarcopenia [21,22]. Therefore, we aimed to determine the proportion of sarcopenia and its associated factors among elderly aged 60 years and above with T2DM in two public primary care clinics in Malaysia.

## Methods

### Ethical consideration

Ethical approval was obtained from the Malaysian Medical Research Ethics Committee, Ministry of Health Malaysia [NMRR-17-1613-36439]. Verbal and written informed consent were obtained from the participants after the researcher explained about the study and the participants had read the participants information sheet. Participation was voluntary. The participants were assigned non-identifiable identification codes for data entry and data analysis. The participants would not be identified in the report writing or publication.

### Data source and participants

This study utilized baseline data of an ongoing longitudinal study–Muscle Strength in Community Living Elderly with Diabetes Mellitus (MUSCLE-DM) study that was registered with the Malaysian National Medical Research Register [Research ID: 36439]. The MUSCLE-DM study focused on the incidence of sarcopenia and its associated factors in two public primary care clinics in Selangor, Malaysia. A total of 506 people with T2DM aged ≥60 years were systematically random sampled (1 in every fifth person) from January to December 2018. Those diagnosed with T2DM for at least one-year duration and on regular follow-up care (≥2 visits in the last 12 months) were included in this study. The diagnosis of T2DM is made by the attending doctor based on either fasting or random venous blood glucose of ≥7.0mmol/L or ≥11.1mmol/L, respectively; or glycated haemoglobin of ≥6.3%, in accordance to the Malaysian guidelines on the Management of Type 2 Diabetes Mellitus, 2015 [23]. Those with conditions which may hinder assessment for sarcopenia such as history of stroke, carpal tunnel syndrome, severe hip or knee osteoarthritis, dysarthria or dysphasia, hearing or visual impairment, and cognitive impairment use of walking aid and living in residential care were excluded.

The present study extracted data from a longitudinal study, which the sample size was calculated using G*Power version 3.1.3 software [24] based on the difference in gait speed over time among older adults aged 65 years and above [5]. The estimated sample size was 506 after taking into consideration a power of 80%, two-sided significance level of 5% with a cluster size of 2 and intra-cluster correlation of 0.05, and 20% loss to follow up. However, in this present study, the outcome was the prevalence of sarcopenia. Therefore, a post-hoc sample size was estimated using the prevalence of sarcopenia of 59.8% in older persons in Malaysia as we do not have the local prevalence of sarcopenia among older people with T2DM [25]. A sample size of 370 was needed for this present study, after taking into consideration of 5% level of significance and 95% confidence interval level.

The data collected in MUSCLE-DM study included socio-demography (age, sex, race, highest level of education, marital status, living arrangement), medical (duration of T2DM, treatment for T2DM, presence of diabetes related complications, comorbid conditions, other current medications) and lifestyle (dietary protein intake, physical activity, smoking, alcohol and use of complementary medicine). In addition, assessments of activities of daily livings (ADLs), instrumental ADLs (IADLs), quality of life, frailty and clinical assessments including measurements of blood pressure, anthropometry (BMI and waist circumference), body compositions (muscle mass, body fat percentage), hand-grip strength, and gait speed were measured. The glycosylated haemoglobin and lipid profile were obtained from the medical records based on the most recent investigation done to reflect the current control.

### Study variables

The data extracted for the present study included: 1) socio-demography; 2) medical and lifestyle information (dietary protein intake, physical activity); 3) functional factors: ADLs and IADLs; and 4) clinical assessments: anthropometry, blood pressure, glycosylated haemoglobin, lipid profile, body compositions, hand-grip strength, and gait speed. In the longitudinal study, the recruitment was conducted through personal communication with the patients by the research assistants on the day of their attendance to the respective clinics. The potential participants were screened by the research assistants to determine the eligibility and safety to participate based on the inclusion and exclusion criteria as described above. The participants who agreed to participate were interviewed using a pre-tested structured questionnaire that was used to collect the baseline data. The clinical assessments were measured following the interviews.

The outcome in this study was sarcopenia, which was determined based on the presence of low handgrip strength (<26 kg for men; <18 kg for women) and/or low gait speed (≤0.8 m/sec) together with low muscle mass (defined as skeletal muscle index <7.0 kg/m2 for men and <5.7 kg/m2 for women) using the cut-off levels recommended by the Asian Working Group for Sarcopenia [4]. We used JAMAR Plus hand dynamometer #563212 to measure the handgrip strength of the dominant hand [26]. The measurement was done with the participant seated with forearm at neutral position, elbow flexed at 90°, and wrist dorsiflexed between 0 and 30° and supported on a table. The average handgrip strength (with two attempts) was used for analysis. The gait speed was based on the measurement of average time taken for the participants to walk along a six-meter distance at their usual walking speed [27]. There were a walk-in and a walk-out phase of 1 meter, before and after the six-meter distance, respectively. A bio-electrical impedance analysis machine (OMRON body composition monitor, model HBF-375) was used to measure body muscle mass. The skeletal muscle index was calculated as body muscle mass divided by the square of body height [4].

Dietary protein intake was based on 24-hour dietary recall. The participants were asked on the number of protein meals a day, which included legumes/lentils, red or white meat, fish, seafood, eggs, nuts/ soy/beans or dairy products (such as milk, cheese, yoghurt).

The level of physical activity was measured using the validated and reliable International Physical Activity Questionnaire short-form (IPAQ-SF)[28,29]. The IPAQ-SF has 7 questions on the physical activity performed in the last 7 days based on the vigorous and moderate activities, walking activity and sitting activity. The level of physical activity was categorised into low, moderate and high. The IPAQ-SF sitting question measures time spent in sedentary activity and is not included as part of any summary score of the physical activity level. The sitting activity is reported as median values and interquartile ranges.

The Katz ADLs questionnaire assesses functional status as a measurement of the patient’s ability to perform ADLs independently [30]. It assesses six functions of bathing, dressing, toileting, transferring, continence, and feeding. The participants scored yes/no for independence in each of the six functions. A score of 5–6 indicates full function, 3–4 indicates moderate impairment, and ≤2 indicates severe functional impairment.

The Lawton IADLs scale assesses independent living skills on eight areas of function[31]. These functions are the ability to use the telephone, shopping, food preparation, housekeeping, laundry, mode of transportation, responsibility for own medications and ability to handle finances. Participants are scored according to their highest level of functioning in each function. A summary score ranges from 0 (low function, dependent) to 8 (high function, independent).

The BMI was categorised as a) underweight: <18.5 kg/m^2^; b) normal: 18.5–22.9 kg/m^2^; c) overweight: 23–27.4 kg/m^2^; and d) obese: ≥27.5 kg/m^2^ [32]. Excessive waist circumference (WC) was considered if ≥90cm for men and ≥80cm for women. The blood pressure (BP) was measured on the day of enrolment, and the glycosylated haemoglobin (HbA1c) and fasting lipid profile (comprised low-density lipoprotein cholesterol (LDL-C), high-density lipoprotein cholesterol (HDL-C) and triglyceride (TG)) were obtained from the medical record based on the latest measurements done in the last 3 months. The HbA1c was analysed using the Bio-Rad D-10 high performance liquid chromatography (Bio-Rad Laboratories, CA, USA) by the clinics’ in-house clinical laboratory. The clinics’ in-house laboratory also analysed the fasting lipid profile using the Beckman DxC800 general chemistry analyzer (Beckman Coulter, Fullerton, CA, USA).

### Data analysis

Data were analysed using Statistical Package for Social science (SPSS) software. Descriptive continuous data were reported as means and standard deviations (SDs) for normally distributed data and as median and interquartile range for non-normally distributed data. Categorical data were reported in percentages and frequencies. Binary logistic regression analyses was performed to determine the predictive factors of sarcopenia for all the study variables except ADLs as 99.6% of the participants were independent. The significant variables from the univariate analyses were selected using an enter method with ≤0.250 significance levels for an addition of the variable to predict the outcome and were included in the multiple logistic regression model. The p-value, adjusted odds ratio and 95% confidence level were reported to determine the strength of contribution from each variable towards the presence of sarcopenia.

## Results

Data were extracted from 506 participants. The mean age of the participants was 67.6±6.8 years and majority were between 60–69 years old (65.7%). Most of the participants were women (60.0%), Indian and others ethnicity (42.5%), married (63.4%), of at least primary school education (73.1%) and living with family and/or others (95.1%) (Table 1).

**Table 1:** Participants socio-demographic characteristics and the sarcopenia status (N = 506).

Factors	Total, n (%)	Presence of sarcopenia, n (%)	No sarcopenia, n (%)
Age			
• 60–69 years	332 (65.7)	73 (22.0)	259 (78.0)
• 70–79 years	147 (29.1)	56 (38.1)	91 (61.9)
• ≥80 years	27 (5.2)	15 (55.6)	12 (44.4)
**Gender**			
• Men	202 (40.0)	69 (34.2)	133 (65.8)
• Women	304 (60.0)	75 (24.7)	229 (75.3)
**Ethnicity**			
• Malay	208 (41.1)	50 (24.0)	158 (76.0)
• Chinese	83 (16.4)	26 (31.3)	57 (68.7)
• Indians and Others	215 (42.5)	68 (31.6)	147 (68.4)
**Marital status**			
• Married	321 (63.4)	89 (27.7)	232 (72.3)
• Not married	185 (36.6)	55 (29.7)	130 (70.3)
**Education level**			
• No formal education and primary school	370 (73.1)	106 (28.6)	264 (71.4)
• Secondary school and higher	136 (26.9)	38 (27.9)	98 (72.1)
**Living arrangement**			
• Living alone	25 (4.9)	8 (32.0)	17 (68.0)
• Living with family/others	481 (95.1)	136 (28.3)	345 (71.7)

Table 2 summarises the clinical and lifestyle profiles, and the functional status of the study participants and the sarcopenia status. The prevalence of elderly with T2DM who had sarcopenia was 28.5% (n = 144). The mean duration of diabetes was 10.0±6.6 years and most had T2DM <10 years of duration (56.3%) and were on oral hypoglycaemic agents alone (64.2%) and 91.7% used insulin sensitizer, which was metformin, as no participants were on thiazolidinediones. About a third were on insulin (35.8%) and 51.0% were on non-insulin sensitizers. The non-insulin sensitizers included sulphonylureas (51.05), alpha glucosidase (1.6%) and dipeptidylpeptidase-4 inhibitors (1.2%). Majority of the participants were on ≥2 anti-diabetic agents (68.8%). More than half of the participants had diabetes complications (56.2%) and 19.6% had two or more complications. Most had diabetes retinopathy (75.7%).

**Table 2:** Participants’ clinical, lifestyle and functional status information and the sarcopenia status (n = 506).

Variables	Total, n (%)	Presence of sarcopenia, n (%)	No sarcopenia, n (%)
**Presence of sarcopenia**		144 (28.5)	362 (71.5)
**Duration of diabetes**			
• <10 years	285 (56.3)	74 (26.0)	211 (74.0)
• ≥10 years	221 (43.7)	70 (31.7)	151 (68.3)
**Types of antidiabetic agents (ADA)**			
• Oral ADA only	326 (64.2)	99 (30.5)	227 (69.5)
• Oral ADA and insulin	153 (30.3)	41 (26.8)	112 (73.2)
• Insulin only	28 (5.5)	4 (14.3)	24 (85.7)
**No. of ADA types**			
• One type	158 (31.2)	39 (24.7)	119 (75.3)
• ≥2 types	348 (68.8)	105 (32.2)	243 (69.8)
**Use of insulin sensitizers**			
• Yes	464 (91.7)	137 (29.5)	327 (70.5)
• No	42 (8.3)	7 (16.7)	35 (83.3)
**Use of insulin**			
• Yes	181 (35.8)	45 (25.9)	136 (75.1)
• No	325 (64.2)	99 (30.5)	226 (69.5)
**Use of non-insulin sensitizers**			
• Yes	258 (51.0)	77 (29.8)	181 (70.2)
• No	248 (49.0)	67 (27.0)	181 (73.0)
**Use of sulphonylureas**			
• Yes	258 (51.0)	77 (29.8)	181 (70.2)
• No	248 (49.0)	67 (27.0)	181 (73.0)
**Use of dipeptidylpeptidase-4 inhibitors**			
• Yes	6 (1.2)	3 (50.0)	3 (50.0)
• No	500 (98.8)	141 (28.2)	359 (71.8)
**Use of alpha-glucosidase**			
• Yes	8 (1.6)	5 (62.5)	3 (37.5)
• No	498 (98.4)	139 (27.9)	359 (72.1)
**Presence of diabetes complication**			
• None	222 (43.8)	63 (28.4)	159 (71.6)
• One complication	185 (36.6)	55 (29.7)	130 (70.3)
• ≥2 complications	99 (19.6)	26 (26.3)	73 (73.7)
**Types of diabetes complications (n = 284)**			
• Retinopathy ◦ Yes ◦ No			
	215 (75.7)	62 (28.8)	153 (71.2)
	69 (24.3)	19 (27.5)	50 (72.5)
• Nephropathy ◦ Yes ◦ No			
	30 (10.6)	10 (33.3)	20 (66.7)
	254 (89.4)	71 (28.0)	183 (72.0)
• Neuropathy ◦ Yes ◦ No			
	126 (44.4)	29 (23.0)	97 (77.0)
	158 (55.6)	52 (32.9)	106 (67.1)
• Peripheral vascular disease			
◦ Yes ◦ No	25 (8.8)	7 (28.0)	18 (72.0)
	259 (91.2)	74 (28.6)	185 (71.4)
• Coronary heart disease			
◦ Yes ◦ No	9 (3.2)	3 (33.3)	6 (66.7)
	275 (96.8)	78 (28.4)	197 (71.6)
**Concurrent hypertension**			
• Yes	484 (95.7)	134 (27.7)	350 (72.3)
• No	22 (4.3)	10 (45.5)	12 (54.5)
**Use of anti-hypertensive agents (n = 484)**			
• None	21 (4.4)	7 (33.3)	14 (66.7)
• One type	140 (28.9)	42 (30.0)	98 (70.0)
• ≥2 types	323 (66.7)	85 (26.3)	238 (73.7)
**Types of anti-hypertensive agents (n = 484)**			
• Angiotensin-Converting Enzyme Inhibitor			
◦ Yes ◦ No	328 (67.8)	99 (30.2)	229 (69.8)
	156 (32.2)	35 (22.4)	121 (77.6)
• Angiotensin II Receptor Blockers			
◦ Yes ◦ No	39 (8.1)	2 (5.1)	37 (94.9)
	445 (91.9)	132 (29.7)	313 (70.3)
• Calcium channel blockers			
◦ Yes ◦ No	310 (64.0)	86 (27.7)	224 (72.3)
	174 (36.0)	48 (27.6)	126 (72.4)
• Beta blockers			
◦ Yes ◦ No	123 (25.4)	28 (22.8)	95 (77.2)
	361 (74.6)	106 (29.4)	255 (70.6)
• Diuretics			
◦ Yes ◦ No	154 (31.8)	39 (25.3)	115 (74.7)
	330 (68.2)	95 (28.8)	235 (71.2)
• Alpha blockers			
◦ Yes ◦ No	9 (1.9)	4 (44.4)	5 (55.6)
	475 (98.1)	130 (27.4)	345 (72.6)
**Concurrent dyslipidaemia**			
• Yes	485 (95.8)	136 (28.0)	349 (72.0)
• No	21 (4.2)	8 (38.1)	13 (61.9)
**Use of lipid lowering agents**			
• Statins	439 (90.5)	121 (27.6)	318 (72.4)
• Fibrates	3 (0.6)	0 (0.0)	3 (100.0)
• None	43 (8.9)	15 (34.9)	28 (65.1)
**No. of medications use**			
• <5 medications	166 (32.8)	59 (35.5)	107 (64.5)
• ≥5 medications	340 (67.2)	85 (25.0)	255 (75.0)
**Current smoking status**			
• Yes	36 (7.1)	10 (27.8)	26 (72.2)
• No	470 (92.9)	134 (28.5)	336 (71.5)
**Alcohol use**			
• Yes	16 (3.2)	5 (31.3)	11 (68.7)
• No	490 (96.8)	139 (28.4)	351 (71.6)
**Complementary and alternative medicine use**			
• Yes, for diabetes	28 (5.5)	7 (25.0)	21 (75.0)
• Yes, but not for diabetes	51 (10.1)	12 (23.5)	39 (76.5)
• No	427 (84.4)	125 (29.3)	302 (70.7)
**Body mass index**			
• Underweight (<18.5 kg/m^2^)	12 (2.4)	11 (91.7)	1 (8.3)
• Normal weight (18.5–22.9 kg/m^2^)	78 (15.4)	57 (73.1)	21 (26.9)
• Overweight (23.0–27.4kg/m^2^)	198 (38.9)	60 (30.3)	138 (69.7)
• Obese (≥27.5kg/m^2^)	219 (43.3)	16 (7.3)	203 (92.7)
**Waist circumference**			
• Normal	37 (7.3)	23 (62.2)	14 (37.8)
• Abnormal	469 (92.7)	121 (25.8)	348 (74.2)
**Glycated haemoglobin (HbA1c)**			
• HbA1c ≤7.0%	197 (38.9)	62 (31.5)	135 (68.5)
• HbA1c 7.1–8.0%	97 (19.2)	25 (25.8)	72 (74.2)
• HbA1c >8.0%	212 (41.9)	57 (26.9)	155 (73.1)
**Blood pressure (BP)**			
• Systolic BP (Mean±SD, mmHg)	138.7±19.1	136.89±20.45	139.40±118.42
• Diastolic BP (Mean±SD, mmHg)	73.3±10.3	71.57±10.75	73.94±10.11
**Lipid profile**			
• LDL-C (Mean±SD, mmol/L)	2.7±1.0	2.76±1.05	2.65±1.02
• HDL-C (Mean±SD, mmol/L) ◦ Men ◦ Women			
		
	1.1±0.3	1.16±0.32	1.13±0.28
	1.3±0.3	1.28±0.28	1.25±0.27
• TG (Mean±SD, mmol/L)	1.6±0.9	1.64±1.16	1.59±0.92
**No. of daily dietary protein intake meals**			
• None	24 (4.7)	11 (45.8)	13 (54.2)
• 1 meal a day	182 (36.0)	44 (24.2)	138 (75.8)
• 2 meals a day	184 (36.4)	54 (29.3)	130 (70.7)
• 3 meals a day	116 (22.9)	35 (30.2)	81 (69.8)
**IPAQ-SF**			
• Low	256 (50.6)	78 (30.5)	178 (69.5)
• Moderate	154 (30.4)	48 (31.2)	106 (68.8)
• High	96 (19.0)	18 (18.8)	78 (81.2)
**Sitting activities**			
• Median±IQR, minutes/day	120.0±120.0	120.0±120.0	120.0±120.0
**Activities of daily livings**			
• Independent (scores of 5–6)	504 (99.6)	142 (28.1)	362 (71.9)
• Moderately dependent (scores of 3–4)	2 (0.4)	2 (100.0)	0 (0.0)
• Dependent (scores of ≤2)	0	0 (0.0)	0 (0.0)
**Instrumental activities of daily livings**			
• Mean±SD	5.48±1.87	5.70±1.76	4.94±2.03

SD = standard deviation, IPAQ-SF = International Physical Activity Questionnaire Short-Form, LDL-C = low density lipoprotein cholesterol, HDL-C = high density lipoprotein cholesterol and TG = triglyceride

Majority of the participants had concurrent hypertension (95.7%) (see Table 2) and of these 95.7% were on anti-hypertensive agents. Most participants were on angiotensin-converting enzyme inhibitors (67.8%) and 66.7% were on ≥2 anti-hypertensive agents. Among those with dyslipidaemia (95.8%), 90.5% were on statins. Majority of the participants were on ≥5 types of medications (67.2%). Majority were non-smoker (92.9%), did not consume alcohol (96.8%) and did not use complementary and alternative medicine (84.4%).

The mean BMI of participants was 27.5±8.1kg/m^2^ with most of them had a BMI of ≥27.5kg/m^2^ (43.3%). Most had abnormal WC (92.7%) with the mean WC for men and women were 99.3±11.5cm and 97.6±10.9cm, respectively. The mean HbA1c was 8.1±2.1% with 41.9% had HbA1c >8.0%. The mean systolic BP and diastolic BP were 138.7±19.1mmHg and 73.3±10.3mmHg, respectively. The mean LDL-C and triglyceride were 2.7±1.0mmol/L and 1.6±0.9 mmol/L, respectively and the mean HDL-C for men and women were 1.1±0.3mmol/L and 1.3±0.3mmol/L, respectively. There were 22.9% participants consumed 3 meals of protein in a day and 50.6% had low level of physical activity. The median time spent on sitting activities in a day was 120.0±IQR120.0 minutes. Majority of the participants were independent in their ADLs (99.6%) and the mean score for IADLs was 5.5±1.9.

Tables 3 and 4 presents the associations between sarcopenia and the socio-demographic, clinical, lifestyle and functional factors using the univariate logistic regression analyses. Age(p<0.001), gender(p = 0.021), Malay ethnicity(p = 0.083), duration of diabetes(p = 0.159), use of insulin sensitizers (p = 0.083), use of insulin(p = 0.182), concurrent hypertension(p = 0.077), number of medications use(p = 0.014), body mass index(p<0.001), waist circumference(p<0.001), physical activity(p = 0.030), and instrumental ADLs(p<0.001) had p-values ≤0.25 and were included in the multiple logistic regression analysis.

**Table 3:** The associations between sarcopenia and socio-demographic factors using univariate logistic regression.

Factors	OR (95%CI)	p-value
**Age**		
• 60–69 years	Ref	
• ≥70 years	2.45 (1.64, 3.64)	<0.001*
**Gender**		
• Men	1.58 (1.07, 2.34)	0.021*
• Women	Ref	
**Ethnicity**		
• Malay	0.68 (0.45, 1.05)	0.083
• Chinese	0.98 (0.57, 1.70)	0.960
• Indians and Others	Ref	
**Marital status**		
• Married	Ref	
• Not married	1.10 (0.74, 1.64)	0.630
**Education level**		
• No formal education and primary school	1.04 (0.67, 1.60)	0.876
• Secondary school and higher	Ref	
**Living arrangement**		
• Living alone	1.19 (0.50, 2.83)	0.688
• Living with family/others	Ref	

**Table 4:** The associations between sarcopenia and clinical, lifestyle and functional factors using univariate logistic regression.

Variables	OR (95%CI)	p-value
**Duration of diabetes**		
• <10 years	Ref	
• ≥10 years	1.32 (0.89, 1.95)	0.159
**Use of insulin sensitizers**		
• Yes	Ref	
• No	0.48 (0.21, 1.10)	0.083
**Use of insulin**		
• Yes	Ref	
• No	1.32 (0.88, 1.99)	0.182
**Use of non-insulin sensitizers**		
• Yes	1.15 (0.78, 1.69)	0.481
• No	Ref	
**Diabetes retinopathy**		
• Yes • No	1.07 (0.58, 1.95)	0.835
	Ref	
**Diabetes nephropathy**		
• Yes • No	1.29 (0.58, 2.89)	0.538
	Ref	
**Diabetes neuropathy**		
• Yes • No	0.61 (0.36, 1.04)	0.680
	Ref	
**Peripheral vascular diseases**		
• Yes • No	0.97 (0.39, 2.42)	0.952
	Ref	
**Coronary heart disease**		
• Yes • No	1.26 (0.31, 5.17)	0.746
	Ref	
**Concurrent hypertension**		
• Yes	0.46 (0.19, 1.09)	0.077
• No	Ref	
**Concurrent dyslipidaemia**		
• Yes	0.63 (0.26, 1.56)	0.633
• No	Ref	
**No. of medications use**		
• <5 medications	1.65 (1.11, 2.47)	0.014
• ≥5 medications	Ref	
**Current smoking status**		
• Yes	0.96 (0.45, 2.05)	0.925
• No	Ref	
**Alcohol use**		
• Yes	1.15 (0.39, 3.36)	0.802
• No	Ref	
**Complementary and alternative medicine use**		
• Yes, for diabetes	0.81 (0.33, 1.94)	0.630
• Yes, but not for diabetes	0.74 (0.38, 1.47)	0.393
• No	Ref	
**Body mass index**		
• Underweight (<18.5 kg/m^2^)	4.05 (0.49, 33.33)	0.193
• Normal weight (18.5–22.9 kg/m^2^)	Ref	
• Overweight (23.0–27.4kg/m^2^)	0.16 (0.09, 0.29)	<0.001*
• Obese (≥27.5kg/m^2^)	0.03 (0.01, 0.06)	<0.001*
**Waist circumference**		
• Normal	Ref	
• Abnormal	0.21 (0.11, 0.42)	<0.001*
**Blood pressure (BP)**		
• Systolic BP (Mean±SD, mmHg)	0.99 (0.98, 1.00)	0.310
• Diastolic BP (Mean±SD, mmHg)	0.98 (0.96, 0.99)	0.820
**Lipid profile**		
• LDL-C (Mean±SD, mmol/L)	1.11 (0.91, 1.36)	0.296
• HDL-C (Mean±SD, mmol/L)	1.18 (0.57, 2.44)	0.651
• TG (Mean±SD, mmol/L)	1.05 (0.85, 1.29)	0.646
**No. of daily dietary protein intake meals**		
• 0–1 meal a day	0.84 (0.51, 1.39)	0.505
• 2 meals a day	0.96 (0.58, 1.59)	0.879
• 3 meals a day	Ref	
**IPAQ-SF**		
• Low	1.89 (1.07, 3.38)	0.030*
• Moderate	1.96 (1.06, 3.63)	0.032*
• High	Ref	
**Sitting activities**		
• Median±IQR, minutes/day	1.00 (0.99, 1.00)	0.824
**Instrumental activities of daily livings**		
• Mean±SD	0.81 (0.73, 0.89)	<0.001*

SD = standard deviation, Insulin sensitizers = metformin, IPAQ-SF = International Physical Activity Questionnaire Short-Form, LDL-C = low density lipoprotein cholesterol, HDL-C = high density lipoprotein cholesterol and TG = triglyceride

The factors that predicted sarcopenia among elderly with T2DM were age ≥70 years (OR = 2.07, 95% CI = 1.24, 3.48, p = 0.006), men (OR = 1.84, 95% CI = 1.12, 3.02, p = 0.017), duration of diabetes ≥ 10 years (OR = 1.85, 95% CI = 1.11, 3.09, p = 0.018), on <5 types of medications (OR = 1.98, 95% CI = 1.17, 3.26, p = 0.011), and low (OR = 2.15, 95% CI = 1.07, 4.35, p = 0.032) and moderate (OR = 2.22, 95% CI = 1.07, 4.66, p = 0.033) levels of physical activity. Not using insulin sensitizers (β = -1.44; OR = 0.24; 95%CI = 0.08, 0.71; p = 0.010) and being overweight/obese were less likely to be associated with sarcopenia (OR = 0.09; 95%CI = 0.05, 0.16; p<0.001) (Table 5). With each unit increase in BMI reduced the risk of sarcopenia by 9.0%. There was no collinearity between the factors associated with sarcopenia.

**Table 5:** Multiple logistic regression analysis on factors associated with sarcopenia among elderly with T2DM.

Factors	Beta	SE	Adjusted Odds ratio	95% CI	P-value
**Age**					
• 60–69 years	Ref				
• ≥70 years	0.73	0.26	2.07	1.24, 3.48	0.006*
**Gender**					
• Men	0.61	0.25	1.84	1.12, 3.02	0.017*
• Women	Ref				
**Ethnicity**					
• Malay	-0.17	0.27	0.84	0.50, 1.42	0.519
• Chinese	-0.29	0.35	0.75	0.38, 1.50	0.414
• Indian and others	Ref				
**Duration of diabetes**					
• <10 years	Ref				
• ≥10 years	0.62	0.26	1.85	1.11, 3.09	0.018*
**Use of insulin sensitizers**					
• Yes	Ref				
• No	-1.44	0.56	0.24	0.08, 0.71	0.010*
**Use of insulin**					
• Yes	Ref				
• No	-0.19	0.28	0.83	0.48, 1.43	0.494
**Concurrent hypertension**					
• Yes	-0.14	0.63	0.87	0.25, 2.96	0.820
• No	Ref				
**No. of medications**					
• <5 medications	0.68	0.27	1.98	1.17, 3.36	0.011*
• ≥5 medications	Ref				
**Body mass index**					
• Normal weight	Ref				
• Underweight	1.56	1.12	4.76	0.53, 42.98	0.163
• Overweight & Obese	-2.43	0.33	0.09	0.05, 0.17	<0.001*
**IPAQ-SF**					
• Low	0.77	0.36	2.15	1.07, 4.35	0.032*
• Moderate	0.80	0.38	2.23	1.07, 4.66	0.033*
• High	Ref				
**Instrumental ADLs**	-0.11	0.07	0.89	0.78, 1.02	0.102

Ref = Reference group, SE = standard error, CI = confidence interval, IPAQ-SF = International Physical Activity Questionnaire Short-Form, ADLs = activities of daily livings

Chi-square (8) = 11.656, p = 0.167; Nagelkerke R^2^ = 0.375

## Discussion

This study examined the prevalence of sarcopenia and its associated factors. Some of the factors that predict sarcopenia such as level of physical activity and body mass index were among the modifiable factors that could be used in developing future strategies to prevent or delay the progression of sarcopenia among elderly with T2DM.

In our study, the prevalence of sarcopenia among elderly with T2DM was about 28% which was comparable to the findings in Singapore at 27.4% by Fung et. al. (2019) [15], as their study also used the same definition for sarcopenia as recommended by the Asian Working Group for Sarcopenia [4]. This was based on the presence of low muscle strength and/or physical performance together with low muscle mass. Further, both Malaysia and Singapore have similar multi-ethnic Asian populations. The prevalence of sarcopenia among people with T2DM in the United States of America (USA) and Korea was reported at 29.3%[13] and 15.7% [14], respectively. In the study from the USA, sarcopenia was determined using a screening questionnaire (SARC-F) [13], which is recommended for case finding [3]. Whereas, the study in Korea, sarcopenia was determined by the muscle mass measured using the dual-energy X-ray absorptiometry, which identified muscle mass but not muscle strength to define sarcopenia[14]. The variation in the prevalences could be attributed to the different tools used to determine sarcopenia. Nevertheless, the revised EWGSOP guideline in 2018 recommends the use of low muscle strength as the primary parameter for sarcopenia as it is the most reliable measure of muscle function [3].

Advanced age was associated with sarcopenia in elderly with T2DM, which concurred to the findings by previous studies [13–15]. The physical and functional decline has been reported to occur with advanced age in elderly with reduced muscle mass and strength [33,34]. In addition, T2DM has been shown to be associated with reduced muscle mass in the elderly [9].

Our study found that being men was associated with sarcopenia. Similarly, Kim et. al. (2010) reported that men with T2DM had decreased lean body mass as compared to men without T2DM with similar body weight [14]. It has been shown that men lost greater muscle mass with advanced age compared to women, even though men have greater skeletal muscle mass [33].

Our study reported that a more prolonged duration of T2DM was associated with sarcopenia. We did not find any study that report similar result. However, this finding could be explained by the effect of T2DM has on the elderly leading to reduced muscle mass with the progression of the condition [9]. We also found that patients on less than 5 medications were associated with sarcopenia when compared to those on polypharmacy (on 5 or more medications). There is no study among T2DM with similar findings. A study on hospitalized elderly found polypharmacy was not associated with sarcopenia [35]. In our study, the low number of medication use may suggest that treatment to targets in the presence of co-morbidities and diabetic complications have not been optimized. However, it is worth to note that number of medications alone may not fully predict the occurrence of sarcopenia as it is complex and a result of multiple predisposing factors.

In contrast to previous study, our study found elderly not using insulin sensitizers were less likely to be associated with sarcopenia. A prospective study showed older men with diabetes on insulin sensitizers such as metformin had less muscle mass loss compared to men with untreated diabetes or with diabetes treated without insulin sensitizers [17]. A possible explanation in our study could be due to that higher proportion of the study participants’ glycaemic control was suboptimal. Hence, despite being on insulin sensitizers, the target to control for their diabetes was not achieved, which could contribute to sarcopenia.

Similar to previous studies on factors associated with sarcopenia in elderly, lower BMI increased the risk of sarcopenia [19,20]. Reduced BMI is a proxy measure for poor nutritional status, which may reflect decreased muscle protein synthesis [36]. In addition, previous study on elderly with sarcopenia in Taiwan has shown that sarcopenia was associated with lack of nutrition [19]. Protein intake was not found to be associated with reduced risk of sarcopenia in the present study. This could be attributed to the protein intake variability in this present study population was very small; hence, the sample size could not adequately detect an association.

Low physical activity among elderly with T2DM was associated with sarcopenia as found by previous study on sarcopenia among the elderly in general [20]. In this 4-years longitudinal study, low physical activity was associated with the development of sarcopenia. Also, a local study found that about a third of people with T2DM did not exercise as recommended [37]. A meta-analysis has shown that engaging in physical activity may significantly prevents the elderly from developing sarcopenia, while physical inactivity is a significant risk factor for sarcopenia [38].

This study is not without limitations. First, it used baseline data from an ongoing longitudinal study, which the causal-effect relationship could not be implied. Second, some of the factors associated with sarcopenia could be bi-directional in relationship. For example, low physical activity may be associated with a higher risk of sarcopenia, but sarcopenia per se could also result in reduced physical activity due to low muscle strength. Third, the questionnaire used to measure dietary protein intake only quantified the number of protein meals but not the amount of protein (in grams) consumed in a day, which may affect the accuracy of quantification. Further, the present study should include protein and caloric intake measurements as these affect the development of sarcopenia. Fourth, the prevalence in this study may have been underestimated by excluding people with comorbidities who are not able to perform the assessments needed to determine the prevalence of sarcopenia in this study. Last, the findings of this study would not be generalized to the Malaysia population at large as the study was conducted in two public primary care clinics in Malaysia.

In conclusion, advanced age, being men, duration of T2DM ≥10 years, on combined OHA and insulin, on <5 types of medications, lower BMI, and low and moderate levels of physical activities were associated with sarcopenia. Increasing physical activity and maintaining a healthy weight could be beneficial in the prevention of sarcopenia among the elderly with T2DM. These could be included in the counselling of T2DM self-management in clinical practice. In addition, the evaluation of sarcopenia could be done at primary care setting not just at the tertiary care setting. However, there is a need for a prospective study to evaluate changes over time on the risk factors of sarcopenia.
